# Collaborative Integrated Navigation for Unmanned Aerial Vehicle Swarms Under Multiple Uncertainties

**DOI:** 10.3390/s25030617

**Published:** 2025-01-21

**Authors:** Le Zhang, Xiaomeng Cao, Mudan Su, Yeye Sui

**Affiliations:** 1 Laboratory of Smart Earth, Beijing Institute of Tracking and Telecommunication Technology, Beijing 100094, China; sumudan2000@163.com (M.S.); yeyesuibittt@163.com (Y.S.); 2 School of Electronics and Information Engineering, Xi’an Jiaotong University, Xi’an 701149, China; 3 School of Electrical and Control Engineering, Shaanxi University of Science and Technology, Xi’an 710021, China; xmcao911@163.com

**Keywords:** UAV swarms, integrated navigation, relative positioning

## Abstract

UAV swarms possess unique advantages in performing various tasks and have been successfully applied across multiple scenarios. Accurate navigation serves as the foundation and prerequisite for executing these tasks. Unlike single UAV localization, swarms enable the sharing and propagation of precise positioning information, which enhances overall swarm localization accuracy but also introduces the issue of uncertainty propagation. To address this challenge, this paper proposes an integrated navigation and positioning method that models, propagates, and mitigates uncertainties. To tackle the issue of uncertainty in information quality caused by outliers in external correction data, a robust integrated navigation method for nonlinear systems is derived based on a normal gamma distribution model. Considering uncertainty propagation, a statistical linearization model for nonlinear systems is developed. Building upon this model, an augmented measurement nonlinear least squares positioning method is applied, achieving further improvements in localization accuracy. Simulation experiments demonstrate that the proposed method, which thoroughly accounts for the effects of multiple uncertainties, can achieve robust tracking and provide relatively accurate positioning results.

## 1. Introduction

Unmanned aerial vehicle (UAV) swarms have emerged as a transformative approach in various applications, such as disaster relief, environmental monitoring, precision agriculture, and military operations [1,2]. Unlike single UAV systems, swarms offer significant advantages in terms of scalability, redundancy, and the ability to perform complex tasks collaboratively. For a UAV swarm to function effectively, precise and reliable navigation is paramount. Navigation enables each UAV to determine its position, maintain formations, and execute coordinated tasks efficiently.

Collaborative navigation is particularly critical in UAV swarms, as it allows individual units to share information, collectively compensate for uncertainties, and enhance the overall reliability of the system. This cooperative framework minimizes the reliance on external infrastructure, making UAV swarms more robust and adaptable to dynamic and challenging environments. At the same time, this cooperative approach enables the entire UAV swarm to achieve absolute positioning as long as a subset of the UAVs in the swarm can receive external correction information. Despite its potential, UAV swarm navigation faces significant challenges, primarily due to various sources of uncertainty that can degrade positioning accuracy and system reliability.

For UAVs capable of receiving external correction information, called the leader UAVs, the onboard inertial navigation system (INS) is corrected using this information. Common types of external correction information include global navigation satellite system (GNSS) signals or visual matching with pre-mapped environments. Ref. [3] emphasizes the importance of utilizing multiple sensors for integrated navigation. Ref. [4] implements a loose coupling of GNSS signals, an INS, and LiDAR data, achieving an INS and LiDAR simultaneous localization and mapping (SLAM) integration. When the GNSS is denied, visual matching methods serve as a crucial means of obtaining external information. Ref. [5] presents an integrated navigation system for UAVs in GNSS-denied environments based on a radar odometry and an enhanced visual odometry. Ref. [6] assists visual–inertial odometry in scale estimation and correction by identifying artificial landmarks. However, these external sources are higly uncertain and prone to large errors due to factors like multipath effects [7,8], signal occlusion, or mismatches in visual features [9], and measurements with large errors are called outliers. Outliers can significantly distort the positioning results if not robustly handled. To address the issues of non-line-of-sight (NLOS) and multipath effects, Ref. [7] proposes a novel hybrid federated fusion framework. Ref. [8], in combination with Monte Carlo simulation, introduces a Kalman filter tunning method. Aiming at the problem of mismatching of single-point feature matching, Ref. [9] proposes a new method utilizing conjugate line segments, feature curve elements, and texture color region segmentation to enhance robustness. Ref. [10] proposes a method for modeling and processing data corrupted with outliers based on a normal gamma distribution; however, it is designed for linear systems.

For UAVs that cannot directly receive external correction information, referred to as follower UAVs, the external correction information can be propagated to them based on measurements made by leader UAVs. However, the self-localization uncertainty of a UAV propagates into the relative positioning estimates, compounding errors across the swarm. To address the 3D positioning problem of UAV swarms, Ref. [11] treats the UAV swarm system as a rigid body and determines the final position of each node by calculating the global minimum, thereby achieving relative positioning. Ref. [12] also proposes a range-only EKF navigation system that integrates IMU data with inter-node distance measurements obtained from onboard sensors to mitigate the growth of position, navigation, and timing errors during GNSS outages. Refs. [13,14] introduce the sigma point belief propagation method to enable information exchange between UAVs. However, the positioning error of leader UAVs propagates during the information transfer, leading to increased uncertainty in positioning, a problem highlighted in [15]. Ref. [3] considers the dependence of positioning accuracy on the distribution and accuracy of leader UAVs in the swarm.

The measurement and localization among UAVs primarily rely on range estimation through communication time, such as ultra wide band (UWB) ranging. Using multiple measurements, the position of the target UAV can typically be estimated using methods like least squares, as exemplified by the classical two-step weighted least squares method in [16]. However, the relationship between ranging measurements and positions is highly nonlinear [17,18], while most existing least squares methods are linear in structure [19]. To apply linear least squares, the nonlinear system is often linearized. This approach has two issues. Firstly, when linearizing the ranging model, the positioning accuracy error of the leader UAV is not considered. Secondly, the final estimate remains a linear function of the measurements, failing to fully utilize the measurement information and mismatching the inherent nonlinear characteristics of the problem. These two issues have not been thoroughly investigated in the existing literature, with only preliminary discussions found in [20].

Three main types of uncertainties faced in cooperative localization and navigation of UAV swarms are summarized as follows:

1. Uncertainty in external correction information for the swarm: This information often contains outliers, significantly affecting the localization accuracy of the leader UAVs;
2. Propagation of leader UAV localization uncertainties: When the leader drones localize the follower drones, their own uncertainties are transmitted;
3. Challenges in handling uncertainties due to high nonlinearity in inter-UAV distance measurements.

To address these uncertainties, this paper considers them comprehensively in UAV swarm cooperative localization and navigation, proposing an integrated modeling and processing method, which extends the filter and model in [3,20], while making the following contributions:

1. To address the issue of outliers in external information, a normal gamma distribution is employed to model measurement noise. A robust filter for integrated navigation based on the normal gamma distribution (RINNG) under tight coupling is derived to model and handle uncertainties in external information, which is presented in Section 3.
2. A statistical linearization model is proposed in Section 4 to further account for the uncertainty in the leader UAV’s positioning. The positioning result of the leader UAV is given by the RINNG in Section 3. This contribution focuses on achieving statistical linearization of nonlinear models for the least squares positioning in contribution 3, while considering the uncertainty of the positioning in contribution 1.
3. To address the issue of inadequate information utilization due to the high nonlinearity of the model, an uncorrelated conversion method is applied to achieve least squares localization with nonlinear structures, which is given in Section 5.

The paper is organized as follows. Section 2 formulates the problem and addresses the motivations of the paper. Section 3 proposes the robust filter for integrated navigation based on the normal gamma distribution. Section 4 proposes the statistical linearization for nonlinear systems considering multiple uncertainties. Section 5 derives the augmented nonlinear least square estimation for collaborative positioning among UAVs. Section 6 shows the simulation results, and Section 7 concludes the paper.

## 2. Problem Formulation

### 2.1. Modeling

**Figure 1:**
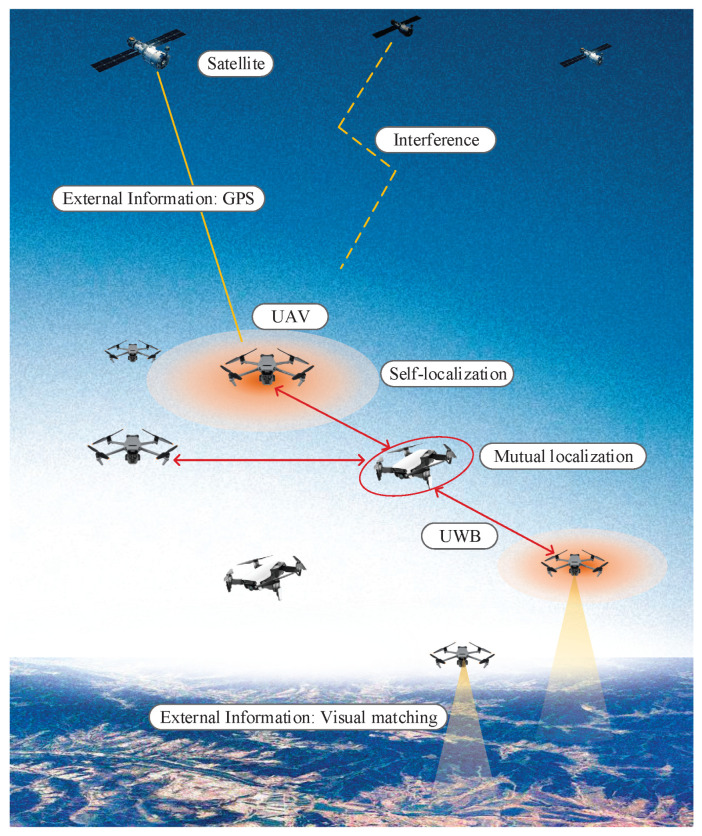
The problem considered in the paper.

Consider a swarm system composed of *N* UAVs, shown in Figure 1, where $n_{1}$ UAVs are equipped with high-precision INSs and/or can receive external correction information, while the remaining $n_{2}$ (i.e., $n_{2}=N-n_{1})$ UAVs are equipped with low-cost, low-precision INS. The available navigation information for the entire swarm includes each UAV’s inertial navigation data, external correction information, and inter-UAV relative positioning data. Our goal is to utilize this information to achieve accurate estimation of each UAV’s dynamic state, with the state defined as

(1) $$x_{k}^{i}={\left[x_{k}^{i},y_{k}^{i},z_{k}^{i}\right]}^{T}$$

where *k* is the time index and *i* the label of the UAV, and ${\left[x_{k}^{i},y_{k}^{i},z_{k}^{i}\right]}^{T}$ denotes the position of the *i*th UAV.

#### 2.1.1. Inertial Navigation System

For the INS, a dynamic system of the system error can be established [1]:

(2) $$\begin{array}{cc} \phi = & \phi \times \omega_{in}^{n}+\delta \omega_{in}^{n}-C_{b}^{n}\varepsilon^{b} \end{array}$$

(3) $$\begin{array}{cc} \delta {\dot{v}}^{n}= & -\phi \times C_{b}^{n}f^{b}+\delta v^{n}\times \left(2\omega_{ie}^{n}+\omega_{en}^{n}\right) \end{array}$$

(4) $$\begin{array}{cc} \; & +v^{n}\times \left(\delta \omega_{ie}^{n}+\delta \omega_{en}^{n}\right)+C_{b}^{n}{▽}^{b} \end{array}$$

(5) $$\begin{array}{cc} \delta \dot{L}= & \frac{\delta v_{N}}{R_{M}+h}-\delta h\frac{v_{N}}{R_{M}+h} \end{array}$$

(6) $$\begin{array}{cc} \delta \dot{\lambda }= & \frac{v_{E}\sec L}{R_{M}+h}+\delta L\frac{v_{E}\tan L\sec L}{R_{N}+h}-\delta h\frac{v_{E}\sec L}{{\left(R_{N}+h\right)}^{2}} \end{array}$$

(7) $$\begin{array}{cc} \delta \dot{h}= & \delta v_{U} \end{array}$$

(8) $$\begin{array}{cc} {\dot{\varepsilon }}_{B}^{b}= & 0 \end{array}$$

(9) $$\begin{array}{cc} {\dot{▽}}_{B}^{b}= & 0 \end{array}$$

Let the inertial navigation velocity $v^{n}={\left[\begin{array}{ccc} v_{E} & v_{N} & v_{U} \end{array}\right]}^{T}$ and the velocity error $\delta v^{n}={\left[\begin{array}{ccc} \delta v_{E} & \delta v_{N} & \delta v_{U} \end{array}\right]}^{T}$, where $v_{E},v_{N}$, and $v_{U}$ represent the components in the east, north, and up directions, respectively. δL,δλ, and δh represent latitude error, longitude error, and altitude error, respectively. $C_{b}^{n}$ denotes the ideal error-free strapdown inertial navigation attitude matrix from the inertial navigation frame to the body coordinate frame. $\delta \omega_{in}^{n}$ is the navigation frame error, with $\delta \omega_{ie}^{n}$ and $\delta \omega_{en}^{n}$ representing the calculation errors of the earth’s rotational angular velocity and the navigation frame’s rotational angular velocity, respectively. ${▽}^{b}$ is the accelerometer measurement bias. $R_{N}$ is the radius of curvature in the prime vertical, and $R_{M}$ is the radius of curvature in the meridian. Let $\Theta_{k}={\left[\phi^{T},{\left(\delta v^{n}\right)}^{T},\delta p,{\left(\varepsilon_{B}^{b}\right)}^{T},{\left({▽}_{B}^{b}\right)}^{T}\right]}^{T}$ with variables $\phi^{T},{\left(\delta v^{n}\right)}^{T},\delta p,{\left(\varepsilon_{B}^{b}\right)}^{T},{\left({▽}_{B}^{b}\right)}^{T}$ being the attitude error, velocity error, position error, gyroscope random drift error, and accelerometer random bias error of the INS, respectively. The dynamic system can be rewritten as

(10) $$\Theta_{k+1}=F_{k}\Theta_{k}+G_{k}w_{k}^{b}$$

where $F_{k}$ is a function of $\Theta_{k}$, $w^{b}={\left[{\left(\varepsilon_{w}^{b}\right)}^{T},{\left({▽}_{B}^{b}\right)}^{T}\right]}^{T}$ is the process noise with $\varepsilon_{w}^{b}$ and ${▽}_{B}^{b}$ being the process noise of the gyroscope random drift and accelerometer components, respectively, and $w_{k}^{b}∼N\left(0,Q_{k}\right)$.

#### 2.1.2. External Correction Information

External correction information refers to navigation data obtained from outside the UAV swarm that can be used to correct the errors in the INS. The most commonly used external correction data is from the GNSS, which provides relatively accurate positioning information to compensate for the cumulative drift of the INS. When the GNSS is unavailable, visual matching becomes another common source of correction information. This method uses onboard cameras to capture images, identify key features, and extract positional data.

Although external correction data are generally assumed to be accurate, outliers often occur in practical applications. The GNSS can experience significant positioning errors under interference, while feature-matching errors in complex environments can also lead to outliers. If these outliers are not properly handled, the corrected positioning error can be substantial, thereby compromising the overall positioning accuracy of the swarm.

Detecting and eliminating these outliers is challenging. On one hand, it is difficult to set a threshold that avoids both missing true outliers and mistakenly discarding valid data. On the other hand, simply discarding potential outliers results in information wasting.

To address the outliers in external correction information, we propose the following measurement modeling method. Assume the position output by the INS is ${\tilde{p}}_{i}$, and the position provided by the external correction information is ${\tilde{p}}_{e}$. Then, the position matching measurement is defined as

(11) $$z={\tilde{p}}_{i}-{\tilde{p}}_{e}$$

and the measurement equation is

(12) $$z_{k}=H_{k}\Theta_{k}+v_{k}$$

with $\Theta_{k}$ being the state defined by (10), and $H_{k}$ the measurement equation defined by

(13) $$H_{k}=\left[0_{3\times 6}\;I_{3\times 6}\;0_{3\times 6}\right]$$

The measurement noise is assumed conditional Gaussian distributed with a probability density function (PDF) given by [10,21]

(14) $$p\left(v_{k}|\tau_{k}\right)=N\left(v_{k};0,\frac{R_{k}}{\tau_{k}}\right)$$

where $\tau_{k}$ is a gamma variable representing the uncertainty in the quality of external correction information with

(15) $$\begin{array}{c} p\left(\tau_{k}\right)=G\left(\tau_{k};a_{k},b_{k}\right)=\frac{b_{k}^{a_{k}}}{\Gamma (b_{k})}\tau_{k}^{a_{k}-1}e^{-b_{k}\tau_{k}} \end{array}$$

The shape parameter of $\tau_{k}$ is denoted by $a_{k}$ and the rate parameter by $b_{k}$. Marginally, the measurement noise $v_{k}$ is Student’s t-distributed, which is a heavy-tailed distribution suitable for modeling measurements with outliers.

#### 2.1.3. Time Difference of Arrival Localization Information

A time difference of arrival (TDOA) measurement is the time difference between a source and a pair of sensors with known positions. To estimate the source position, multiple TDOA measurements are needed. In the considered problem, $n_{1}$ UAVs equipped with high-precision INSs are regarded as sensors to perform TDOA positioning for the remaining $n_{2}$ UAVs with low-precision INSs. The TDOA measurements for the *i*th UAV are

(16) $$Z_{k}^{i}=h\left(x_{k}^{i},X_{k}^{j}\right)+{\overset{ˇ}{V}}_{k}^{i}$$

where $Z_{k}^{i}\in R^{n_{1}-1}$, measurement noise ${\overset{ˇ}{V}}_{k}^{i}\in R^{n_{1}-1}$, the set of locations of sensors $X_{k}^{j}={\left\{x_{k}^{j}\right\}}_{j=1}^{n_{1}}$, and for $j=1,\ldots ,n_{1},i=n_{1}+1,\ldots ,N$

(17) $$Z_{k}^{i}=\left[\begin{array}{c} z_{21}^{i}\\ \vdots \\ z_{n_{1}1}^{i} \end{array}\right]=\left[\begin{array}{c} d_{2}^{i}-d_{1}^{i}\\ \vdots \\ d_{n_{1}}^{i}-d_{1}^{i} \end{array}\right],{\overset{ˇ}{V}}_{k}^{i}=\left[\begin{array}{c} v_{21}^{i}\\ \vdots \\ v_{n_{1}1}^{i} \end{array}\right]$$

(18) $$h\left(x_{k}^{i},X_{k}^{j}\right)=\left[\begin{array}{c} ∥x_{k}^{i}-x_{k}^{2}∥-∥x_{k}^{i}-x_{k}^{1}∥\\ \vdots \\ ∥x_{k}^{i}-x_{k}^{n_{1}}∥-∥x_{k}^{i}-x_{k}^{1}∥ \end{array}\right]$$

(19) $$\begin{array}{c} d_{j}^{i}=∥x_{k}^{i}-x_{k}^{j}∥=\sqrt{{\left(x_{k}^{i}-x_{k}^{i}\right)}^{2}+{\left(y_{k}^{i}-y_{k}^{j}\right)}^{2}+{\left(z_{k}^{i}-z_{k}^{i}\right)}^{2}} \end{array}$$

Without lose of generality, we select UAV 1 as the reference. The arrival times at all other UAVs are substracted from the arrival time at UAV 1 $x_{k}^{1}$ to obtain the TDOA measurement in Equation (18). Note that unlike most TDOA problems, in Equation (18), h(·) is not only the function of $x_{k}^{i}$, but also of $x_{k}^{j},j=1,\ldots ,n_{1}$, and they are also random. The uncertainty of $x_{k}^{j}$ further influences the estimation of $x_{k}^{i}$. Additionally, h(·) is highly nonlinear. All these factors make it extremely challenging to use UAVs equipped with high-precision INSs to locate other UAVs.

### 2.2. Motivations

Collaborative integrated navigation in UAV swarms enables the effective fusion of various sensor information and the transfer of high-precision navigation data from a set of UAVs to the entire swarm, thereby enhancing overall positioning accuracy. However, this approach faces significant challenges due to various uncertainties.

First, there is the uncertainty in external correction information. Common sources of external correction include the GNSS and visual matching. While the GNSS provides high accuracy, it is susceptible to interference and may be denied in highly contested environments, rendering it unable to deliver correction data. Moreover, adversaries could intercept and retransmit fake GNSS signals to deceive the system. Visual matching, on the other hand, is less affected by electromagnetic interference. If terrain features or key landmarks [6] are accurately matched, it can offer precise correction information. However, matching errors and offsets frequently occur, leading to significant errors in correction data at certain times. Both the GNSS and visual matching share a common issue: the correction information they provide often contains numerous outliers. If these outliers are not properly handled, they can cause integrated navigation to fail, which would further compromise the positioning accuracy of the entire swarm. Simply identifying and removing outliers carries the risk of false positives or missed detections, resulting in information loss. Therefore, a more effective solution is to accurately model measurements with outliers for better integrated navigation.

The most popular approach to handling measurement outliers is to model measurement noise using heavy-tailed distributions. The core idea is to assign higher probabilities to large noise values. Common heavy-tailed distributions include the t-distribution and its variants whose marginal distributions are also t-distributed [21,22,23,24]. In this paper, a conditional Gaussian distribution is proposed, whose marginal distribution is also a t-distribution, effectively capturing measurements with outliers. Compared to existing modeling methods, this approach is simpler and facilitates the development of a closed-form filter within the Bayesian framework.

Secondly, mutual positioning among UAVs enables the transfer of high-precision location information but also propagates the uncertainties in positioning results. With the support of a high-precision INS and external correction information, some UAVs in the swarm achieve relatively accurate self-positioning, while others maintain lower self-positioning accuracy. UAVs with accurate self-positioning can measure the positions of other UAVs, facilitating mutual positioning within the swarm and spreading high-precision information throughout. However, since the self-positioning of high-precision UAVs is still based on the estimate that is a function of the external correction data, it inherently contains uncertainty. Additionally, outliers in the external correction information further exacerbate this uncertainty. If the impact of these uncertainties is ignored and a conventional TDOA positioning method is directly applied, it can result in significant positioning errors.

Thirdly, the TDOA measurements are highly nonlinear with respect to the estimand, making TDOA-based positioning essentially a nonlinear estimation problem. Traditional TDOA algorithms primarily rely on the least squares method. However, the existing least squares methods provide a linear estimator of the measurements, meaning they are linear functions of the measurements, which is mismatched with the inherently nonlinear nature of the estimation problem. The limitation imposed by the linear structure of the estimator makes it difficult to improve the accuracy of the positioning results. Under conditions of strong nonlinearity, modeling, describing, and processing the uncertainties in both external correction information and self-localization become even more challenging. This has prompted us to propose new, more effective methods for extracting positioning information from nonlinear systems.

**Figure 2:**
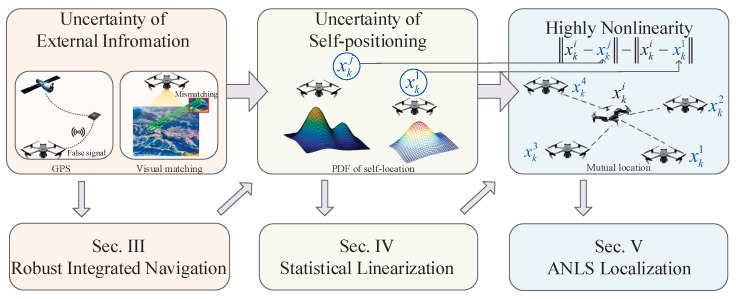
Motivations of this paper.

In summary, in collaborative positioning for UAV swarms, the first step is to achieve high-precision self-localization for some UAVs through integrated navigation using external correction information and INSs, with a focus on addressing outliers in the correction data. Secondly, based on the self-localization results and considering their uncertainties, TDOA positioning is performed for the remaining UAVs, emphasizing the propagation of self-localization uncertainties and the development of a nonlinear TDOA method. The motivations of this paper are summarized by Figure 2.

## 3. Robust Filter for Integrated Navigation Based on the Normal Gamma Distribution

According to Figure 2, external correction information and INSs are first used for integrated navigation to determine the positions of a set of UAVs. For the GNSS, interference may cause only a subset of UAVs to receive satellite signals. For visual matching, limitations in observation angles may result in only some UAVs successfully matching to positioning features. Both types of information may contain outliers, necessitating a robust integrated navigation method to handle the uncertainties in the correction data while also accounting for the uncertainties in the self-localization results. The following proposes such a robust filter to address the uncertainties, which is the filter mentioned by contribution 1, and is named the robust filter for integrated navigation based on the normal gamma distribution (RINNG). The models of integrated navigation are defined by (10) and (12), and summarized as follows.

(20) $$\left\{\begin{array}{c} \Theta_{k+1}=F_{k}\Theta_{k}+G_{k}w_{k}^{b}\\ z_{k}=H_{k}\Theta_{k}+v_{k} \end{array}\right.$$

with $w_{k}^{b}$ and $v_{k}$ defined by (10) and (14), respectively.

Assume that at time k−1, the state $\Theta_{k-1}$ and $\tau_{k-1}$ are jointly distributed with PDF $p\left(\Theta_{k-1},\tau_{k-1}|Z^{k-1}\right)=\mathrm{NG}\left(\Theta_{k-1},\tau_{k-1};{\hat{x}}_{k-1|k-1},P_{k-1|k-1},{\hat{a}}_{k-1|k-1},{\hat{b}}_{k-1|k-1}\right)$, where $\tau_{k-1}$ is the uncertainty variable.

### 3.1. Time Update

The predicted filtering density at time *k* can be obtained by the Chapman–Kolmogorov equation based on the prior density,

(21) $$\begin{array}{cc} \; & p(\Theta_{k},\tau_{k}|z^{k-1})\\ = & \;\int \int \;p\left(\Theta_{k}|\Theta_{k-1}\right)p\left(\tau_{k}|\Theta_{k-1},\tau_{k-1}\right)p\left(\Theta_{k-1},\tau_{k-1}|Z^{k-1}\right)d\tau_{k-1}d\Theta_{k-1}\\ = & \int p\left(\Theta_{k}|\Theta_{k-1}\right)p\left(\Theta_{k-1},\tau_{k}|z^{k-1}\right)d\;\Theta_{k-1} \end{array}$$

where

(22) $$\begin{array}{c} p\left(\Theta_{k-1},\tau_{k}|z^{k-1}\right)=\int p\left(\tau_{k}|\Theta_{k-1},\tau_{k-1}\right)p\left(\Theta_{k-1},\tau_{k-1}|z^{k-1}\right)d\tau_{k-1}. \end{array}$$

Assume that the evolution of $\Theta_{k}$ is independent of $\tau_{k}$ and $\tau_{k-1}$, and then we have

(23) $$p\left(\Theta_{k}|\Theta_{k-1}\right)=N\left(x_{k};F_{k}\Theta_{k-1},Q_{k}\right)$$

By (22), the density $p(\tau_{k}|\Theta_{k-1},\tau_{k-1})$ is needed to get $p(\Theta_{k-1},\tau_{k}|z^{k-1})$. However, it is not straightforward to have a $p(\tau_{k}|\Theta_{k-1},\tau_{k-1})$ such that (22) is a normal gamma distribution. We follow the idea of [21,22,23,24] to obtain the predicted density $p(\Theta_{k-1},\tau_{k}|z^{k-1})$ by replacing ${\hat{a}}_{k-1|k-1}$ and ${\hat{b}}_{k-1|k-1}$ in $p(\Theta_{k-1},\tau_{k-1}|z^{k-1})$ with ${\hat{a}}_{k|k-1}$ and ${\hat{b}}_{k|k-1}$:

(24) $$\begin{array}{cc} \; & p(\Theta_{k-1},\tau_{k}|z^{k-1})\\ = & N\left(\Theta_{k-1};{\hat{\Theta }}_{k-1|k-1},P_{k-1|k-1}/\tau_{k}\right)G\left(\tau_{k};{\hat{a}}_{k|k-1},{\hat{b}}_{k|k-1}\right)\\ = & \mathrm{NG}(\Theta_{k-1},\tau_{k};{\hat{\Theta }}_{k-1|k-1},P_{k-1|k-1},{\hat{a}}_{k|k-1},{\hat{b}}_{k|k-1}) \end{array}$$

where

(25) $$\begin{array}{cc} {\hat{a}}_{k|k-1} & =\rho {\hat{a}}_{k-1|k-1} \end{array}$$

(26) $$\begin{array}{cc} {\hat{b}}_{k|k-1} & =\rho {\hat{b}}_{k-1|k-1} \end{array}$$

with ρ∈(0,1]. By (25) and (26), the predicted density of $\tau_{k}$ is

(27) $$p\left(\tau_{k}|Z^{k-1}\right)=G\left(\tau_{k};{\hat{a}}_{k|k-1},{\hat{b}}_{k|k-1}\right)$$

One advantage of this technique is that it keeps ${\hat{\tau }}_{k|k-1}$ identical to ${\hat{\tau }}_{k-1|k-1}$, and just scales its variance up by $\rho^{-1}$ since the variance of $\tau_{k}$ is $a_{k}/b_{k}^{2}$, which means that without new information, the estimate becomes more uncertain. This is natural when we indeed have no knowledge of the evolution. The value ρ=1 corresponds to stationary noise, and a lower value (0<ρ<1) increases the time fluctuation. More discussions on this technique can be found in [21,22,23,24].

Substituting (23) and (27) into (21) yields

(28) $$\begin{array}{c} p\left(\Theta_{k},\tau_{k}|z^{k-1}\right)=N\left(\Theta_{k};{\bar{\Theta }}_{k|k-1},{\bar{P}}_{k|k-1}/\tau_{k}\right)G\left(\tau_{k};{\hat{a}}_{k|k-1},{\hat{b}}_{k|k-1}\right) \end{array}$$

The following derivation shows how to get Equation (28), including ${\bar{\Theta }}_{k|k-1}$ and ${\bar{P}}_{k|k-1}$.

Rewrite $p(\Theta_{k}|\Theta_{k-1})$ as

(29) $$\begin{array}{cc} p(\Theta_{k}|\Theta_{k-1})\propto & \exp[-\frac{1}{2}{\left(\Theta_{k}-F_{k}\Theta_{k-1}\right)}^{T}Q_{k}^{-1}\left(\cdot \right)] \end{array}$$

(30) $$\begin{array}{cc} \propto & \exp[-\frac{\tau_{k}}{2}{\left(\Theta_{k}-F_{k}\Theta_{k-1}\right)}^{T\;}{\left(\tau_{k}Q_{k}\right)}^{-1}\left(\cdot \right)] \end{array}$$

By (30), Equation (28) becomes

(31) $$\begin{array}{cc} \; & \int_{R^{n_{x}}}\exp\left[-\frac{\tau_{k}}{2}{\left(\Theta_{k}-F_{k}\Theta_{k-1}\right)}^{T}{\left(\tau_{k}Q_{k}\right)}^{-1}\left(\cdot \right)\right]\\ \; & \times \tau_{k}^{n_{x}/2+{\hat{a}}_{k|k-1}-1}\exp\{-\frac{\tau_{k}}{2}[2{\hat{b}}_{k|k-1}\\ \; & +{\left(\Theta_{k-1}-{\hat{\Theta }}_{k-1|k-1}\right)}^{T}P_{k-1|k-1}^{-1}\left(\cdot \right)]\}dx_{k-1}\\ = & \tau_{k}^{n_{x}/2+{\hat{a}}_{k|k-1}-1}\exp\left(-\tau_{k}{\hat{b}}_{k|k-1}\right)\\ \; & \times \int_{R^{n_{x}}}\exp\{-\frac{\tau_{k}}{2}[{\left(\Theta_{k}-F_{k}\Theta_{k-1}\right)}^{T}{\left(\tau_{k}Q_{k}\right)}^{-1}\left(\cdot \right)\\ \; & +{\left(\Theta_{k-1}-{\hat{\Theta }}_{k-1|k-1}\right)}^{T}P_{k-1|k-1}^{-1}\left(\cdot \right)]\}d\Theta_{k-1} \end{array}$$

Note that $F_{k}$ is a function of $\Theta_{k-1}$, making Equation (31) an integral of a nonlinear function multiplied by a Gaussian distribution. This form is well suited for approximating state prediction using Gauss–Hermite quadrature [25], which is a fixed-point sampling method. The basic steps are as follows:

1. The integration points $\chi_{m},m=1,\ldots ,M$ are generated, where *M* is a predetermined number, usually chosen as 3 or 5. Construct a diagonal symmetric matrix *D* with elements on the diagonal being 0 and $D_{m,m+1}=\sqrt{m/2},m=1,\ldots ,M-1$. Calculate the eigenvalues of the matrix *D*, and let the integration points $\chi_{m}=\sqrt{2}\lambda_{m}$ with $\lambda_{m}$ being the *m*th eigenvalue. Let the normalized eigenvector corresponding to $\lambda_{m}$ be denoted as $\nu_{m}$, then the weight $\omega_{m}={\left(\nu_{m}^{1}\right)}^{2}$, where $\nu_{m}^{1}$ is the first element of $\nu_{m}$.
2. Based on the estimate ${\hat{\Theta }}_{k-1|k-1}$ and its MSE $P_{k-1|k-1}$, sigma points are generated, with the *m*th sigma point being
  (32) $$\xi_{m}=\sqrt{P_{k-1|k-1}}\chi_{m}+{\hat{\Theta }}_{k-1|k-1}$$
  where $\sqrt{P_{k-1|k-1}}$ is the result of the Cholesky decomposition of $P_{k-1|k-1}$, i.e., $P_{k-1|k-1}=$$\sqrt{P_{k-1|k-1}}{\sqrt{P_{k-1|k-1}}}^{T}$.
3. Based on the sigma points and their weights, the conditional mean is
  (33) $${\bar{\Theta }}_{k|k-1}=E\left[\Theta_{k}|\tau_{k},z^{k-1}\right]=\sum_{m=1}^{M}\omega_{m}F_{k}\left(\xi_{m}\right)\xi_{m}$$
  and the conditional MSE matrix is
  (34) $$\begin{array}{c} C_{k|k-1}=E\left[\left(\Theta_{k}-{\bar{\Theta }}_{k|k-1}\right){\left(\Theta_{k}-{\bar{\Theta }}_{k|k-1}\right)}^{T}|\tau_{k},z^{k-1}\right]={\bar{P}}_{k|k-1}/\tau_{k} \end{array}$$
  where the conditional scale matrix ${\bar{P}}_{k|k-1}$ is
  (35) $${\bar{P}}_{k|k-1}=\sum_{m=1}^{M}\omega_{m}\left(F_{k}\left(\xi_{m}\right)\xi_{m}-{\bar{\Theta }}_{k|k-1}\right){\left(\cdot \right)}^{T}+\tau_{k}Q_{k}={\overset{ˇ}{P}}_{k|k-1}+\tau_{k}Q_{k}$$

Therefore, the predicted density is

(36) $$N\left(\Theta_{k};{\bar{\Theta }}_{k|k-1},{\bar{P}}_{k|k-1}/\tau_{k}\right)G\left(\tau_{k};{\hat{a}}_{k|k-1},{\hat{b}}_{k|k-1}\right)$$

We desire the predicted density $p(\Theta_{k},\tau_{k}|Z^{k-1})$ to have a normal gamma form, which requires that its mean and scale matrix do not depend on $\tau_{k}$. Since Equations (34) and (35) depend on $\tau_{k}$, the current density (36) is not a desirable predicted density. To get such a $p(\Theta_{k},\tau_{k}|z^{k-1}),$ we choose a normal gamma distribution that is closest to the predicted density (36). Denote the density (36) as $q(\Theta_{k},\tau_{k}|z^{k-1})$. We would like to find a $p(\Theta_{k},\tau_{k}|z^{k-1})$ whose dissimilarity with $q(\Theta_{k},\tau_{k}|z^{k-1})$ is minimized. Assume that such a normal gamma density has the following form,

(37) $$p\left(\Theta_{k},\tau_{k}|z^{k-1}\right)=\mathrm{NG}\left(\Theta_{k},\tau_{k};{\hat{\Theta }}_{k|k-1},P_{k|k-1}^{m},{\hat{a}}_{k|k-1},{\hat{b}}_{k|k-1}\right)$$

where

(38) $$P_{k|k-1}^{m}={\overset{ˇ}{P}}_{k|k-1}+m_{k}Q_{k}$$

with a positive constant $m_{k}>0$ to be determined. Note that $p(\Theta_{k},\tau_{k}|Z^{k-1})$ differs from $q(\Theta_{k},\tau_{k}|Z^{k-1})$ only in the scale matrix $P_{k|k-1}^{m}$.

We adopt the K-L divergence

(39) $$D_{\mathrm{KL}}[q\left(\Theta_{k},\tau_{k}|z^{k-1}\right)||p\left(\Theta_{k},\tau_{k}|z^{k-1}\right)]$$

as the optimality criterion that measures information loss by using $p(\Theta_{k},\tau_{k}|Z^{k-1})$ to approximate $q(\Theta_{k},\tau_{k}|z^{k-1})$. That is, we find a density in the form of (37) that is closest to $q(\Theta_{k},\tau_{k}|Z^{k-1})$ (36) in the K-L sense:

(40) $$p\left(\Theta_{k},\tau_{k}|z^{k-1}\right)=\underset{p}{\arg\min}D_{\mathrm{KL}}\left(q||p\right)$$

with the parameter $m_{k}$ of $p(\Theta_{k},\tau_{k}|Z^{k-1})$ to be determined. The minimum point of $D_{\mathrm{KL}}\left(q||p\right)$ in (40) is $m_{k}=$ E$\left[\tau_{k}|Z^{k-1}\right]={\hat{\tau }}_{k|k-1}.$ Note that ${\hat{\tau }}_{k|k-1}={\hat{\tau }}_{k-1|k-1}$. This ensures that the order of predicting $\Theta_{k}$ and $\tau_{k}$ does not change the results.

### 3.2. Measurement Update

The posterior density can be obtained as follows, since the measurement model is linear:

(41) $$p\left(\Theta_{k},\tau_{k}|z^{k}\right)=\frac{p\left(z_{k}|\Theta_{k},\tau_{k}\right)p\left(\Theta_{k},\tau_{k}|z^{k-1}\right)}{p(z_{k}|z^{k-1})}$$

The likelihood function is

(42) $$\begin{array}{c} p\left(z_{k}|\Theta_{k},\tau_{k}\right)=N\left(z_{k};H_{k}\Theta_{k},\frac{R_{k}}{\tau_{k}}\right)\propto \tau_{k}^{n_{x}/2}\exp\left[-\frac{\tau_{k}}{2}{\left(z_{k}-H_{k}x_{k}\right)}^{T}R_{k}^{-1}\left(\cdot \right)\right] \end{array}$$

Since the normal gamma distribution is a conjugate prior of the normal distribution, the posterior density is still a normal gamma distribution, given by

(43) $$\begin{array}{cc} \; & p(\Theta_{k},\tau_{k}|z^{k})\\ = & \mathrm{NG}(\Theta_{k},\tau_{k};{\hat{\Theta }}_{k|k},P_{k|k},{\hat{a}}_{k|k},{\hat{b}}_{k|k})\\ \propto & \tau_{k}^{n_{z}/2+{\hat{a}}_{k|k}-1}\exp\left\{-\frac{\tau_{k}}{2}\left[2{\hat{b}}_{k|k}+{\left(\Theta_{k}-{\hat{\Theta }}_{k|k}\right)}^{T}P_{k|k}^{-1}\left(\cdot \right)\right]\right\} \end{array}$$

with

(44) $$\begin{array}{cc} {\hat{\Theta }}_{k|k} & ={\hat{\Theta }}_{k|k-1}+K_{k}\left(z_{k}-H_{k}{\hat{\Theta }}_{k|k-1}\right) \end{array}$$

(45) $$\begin{array}{cc} P_{k|k} & =P_{k|k-1}-K_{k}S_{k}K_{k}^{T} \end{array}$$

(46) $$\begin{array}{cc} {\hat{a}}_{k|k} & ={\hat{a}}_{k|k-1}+\frac{n_{z}}{2} \end{array}$$

(47) $$\begin{array}{cc} {\hat{b}}_{k|k} & ={\hat{b}}_{k|k-1}+\left[{\left(z_{k}-H_{k}{\hat{\Theta }}_{k|k}\right)}^{T}R_{k}^{-1}\left(\cdot \right)+{\left({\hat{\Theta }}_{k|k-2}-{\hat{\Theta }}_{k|k}\right)}^{T}P_{k|k-1}^{-1}\left(\cdot \right)\right]/2 \end{array}$$

where

(48) $$\begin{array}{cc} S_{k} & =R_{k}+H_{k}P_{k|k-1}H_{k}^{T} \end{array}$$

(49) $$\begin{array}{cc} K_{k} & =P_{k|k-1}H_{k}S_{k}^{-1} \end{array}$$

The posterior density of $\Theta_{k}$ conditioned on $\tau_{k}$ is Gaussian,

(50) $$p\left(\Theta_{k}|\tau_{k},z^{k}\right)=N\left(\Theta_{k};{\hat{\Theta }}_{k|k},\frac{P_{k|k}}{\tau_{k}}\right)$$

and, marginally, $\Theta_{k}$ is t-distributed

(51) $$p\left(\Theta_{k}|z^{k}\right)=T\left(\Theta_{k};{\hat{\Theta }}_{k|k},\frac{{\hat{b}}_{k|k}}{{\hat{a}}_{k|k}}P_{k|k},2{\hat{a}}_{k|k}\right)$$

Equations (50) and (51) indicate that the posterior distribution of $\Theta_{k}$ accounts for the influence of the variable $\tau_{k}$, which represents the uncertainty in the external correction information.

Based on the INS solution ${\hat{x}}_{k}^{j,\mathrm{INS}}$ at time *k* and the estimate of $\Theta_{k}$, the location of $x_{k}^{j}$ is given by

$$\begin{array}{cc} {\hat{x}}_{k|k}^{j}= & {\hat{x}}_{k}^{j,\mathrm{INS}}+{\hat{\Theta }}_{k|k}^{7:9}\\ P_{k|k}^{j}= & P_{k|k}^{7:9} \end{array}$$

where ${\hat{\Theta }}_{k|k}^{7:9}$ refers to the 7th to 9th dimensions of ${\hat{\Theta }}_{k|k}$, and $P_{k|k}^{7:9}$ the 7th to 9th rows and columns of the matrix $P_{k|k}$.

## 4. Statistical Linearization for Nonlinear Systems Considering Multiple Uncertainties

After completing the self-localization of some UAVs to get their location estimates ${\hat{X}}_{k|k}={\left\{{\hat{x}}_{k|k}^{j}\right\}}_{j=1}^{n_{1}}$, these UAVs are treated as base stations. Through methods such as UWB, inter-UAV ranging is performed to obtain TDOA measurements. In Equation (16), h(·) is a nonlinear function, $x_{k}^{i}$ represents the position of the UAV to be estimated, $x_{k}^{j}$, $j=1,\ldots ,n_{1}$ denote the positions of the self-localized UAVs (base stations), and ${\overset{ˇ}{V}}_{k}^{i}$ is the measurement error with a mean of E$\left[{\overset{ˇ}{V}}_{k}^{i}\right]$ and covariance of $C_{\overset{ˇ}{V}}$. Generally, E$[{\overset{ˇ}{V}}_{k}^{i}]=0.$ For the nonlinear estimation problem described by Equation (16), to estimate the position $x_{k}^{i}$ using the least squares method, the system must first be linearized, resulting in the following approximate system,

(52) $$h\left(x_{k}^{i},X_{k},{\overset{ˇ}{V}}_{k}^{i}\right)\approx L_{h}\left(x_{k}^{i},X_{k},V_{k}^{i}\right)=b_{k}+H_{k}\left(x_{k}^{i}-{\bar{x}}_{k}^{i}\right)+V_{k}^{i}$$

where $b_{k}$ and $H_{k}$ are the parameters to be determined. Note that $V_{k}$ and ${\overset{ˇ}{V}}_{k}$ are different: $V_{k}$ represents the measurement noise after accounting for multiple uncertainties. These uncertainties include the uncertainty in external correction information, the resulting uncertainty in UAV self-localization $X_{k}$, and the uncertainty in TDOA measurements, i.e., ${\overset{ˇ}{V}}_{k}^{i}$.

To determine the above parameters, the following two assumptions are considered [20]:

1. The function h(·) is continuously differentiable and can be sufficiently approximated by a first-order Taylor series expansion in the neighborhood of a given point.
2. The first two moments of $L_{h}\left(\cdot \right)$ and h(·) are equal.

Based on the above two assumptions, the statistical linearized system can be obtained,

(53) $$L_{h}\left(x_{k}^{i},X_{k},V_{k}\right)=h\left({\bar{x}}_{k}^{i},{\hat{X}}_{k|k},0\right)+H_{k}\left(x_{k}-{\bar{x}}_{k}^{i}\right)+V_{k}$$

where $H_{k}={\left.\frac{\partial h(x_{k}^{i},X_{k},{\overset{ˇ}{V}}_{k}^{i})}{\partial x_{k}}\right|}_{x_{k}^{i}={\bar{x}}_{k}^{i},X_{k}={\hat{X}}_{k|k},{\overset{ˇ}{V}}_{k}^{i}=0}$ with ${\hat{X}}_{k|k}={\left\{{\hat{x}}_{k|k}^{j}\right\}}_{j=1}^{n_{1}}$. The first two moments of $V_{k}$ can be obtained by deterministic sampling [25,26] based on

(54) $$\begin{array}{cc} E[V_{k}]= & E[h\left({\bar{x}}_{k}^{i},X_{k},{\overset{ˇ}{V}}_{k}^{i}\right)-h\left({\bar{x}}_{k}^{i},{\hat{X}}_{k|k},0\right)] \end{array}$$

(55) $$\begin{array}{cc} \mathrm{Cov}[V_{k}]= & E[\left\{h\left({\bar{x}}_{k}^{i},X_{k},{\overset{ˇ}{V}}_{k}^{i}\right)-h\left({\bar{x}}_{k}^{i},{\hat{X}}_{k|k},0\right)\right\}{\left\{\cdot \right\}}^{T}] \end{array}$$

and be calculated by

(56) $$\begin{array}{cc} E[V_{k}]\approx & \sum_{m=1}^{M}\varpi_{m}\left[h\left({\bar{x}}_{k}^{i},X_{k}^{m},{\overset{ˇ}{V}}_{k}^{i,m}\right)-h\left({\bar{x}}_{k}^{i},{\hat{X}}_{k|k},0\right)\right] \end{array}$$

(57) $$\begin{array}{cc} \mathrm{Cov}[V_{k}]\approx & \sum_{m=1}^{M}\varpi_{m}\left[h\left({\bar{x}}_{k}^{i},X_{k}^{m},{\overset{ˇ}{V}}_{k}^{i,m}\right)-h\left({\bar{x}}_{k}^{i},{\hat{X}}_{k|k},0\right){\left(\cdot \right)}^{T}\right] \end{array}$$

where $X_{k}^{m}$ and ${\overset{ˇ}{V}}_{k}^{i,m}$ are the samples of $X_{k}$ and ${\overset{ˇ}{V}}_{k}^{i}$, and $\varpi_{m}$ is the corresponding weight.

**Figure 3:**
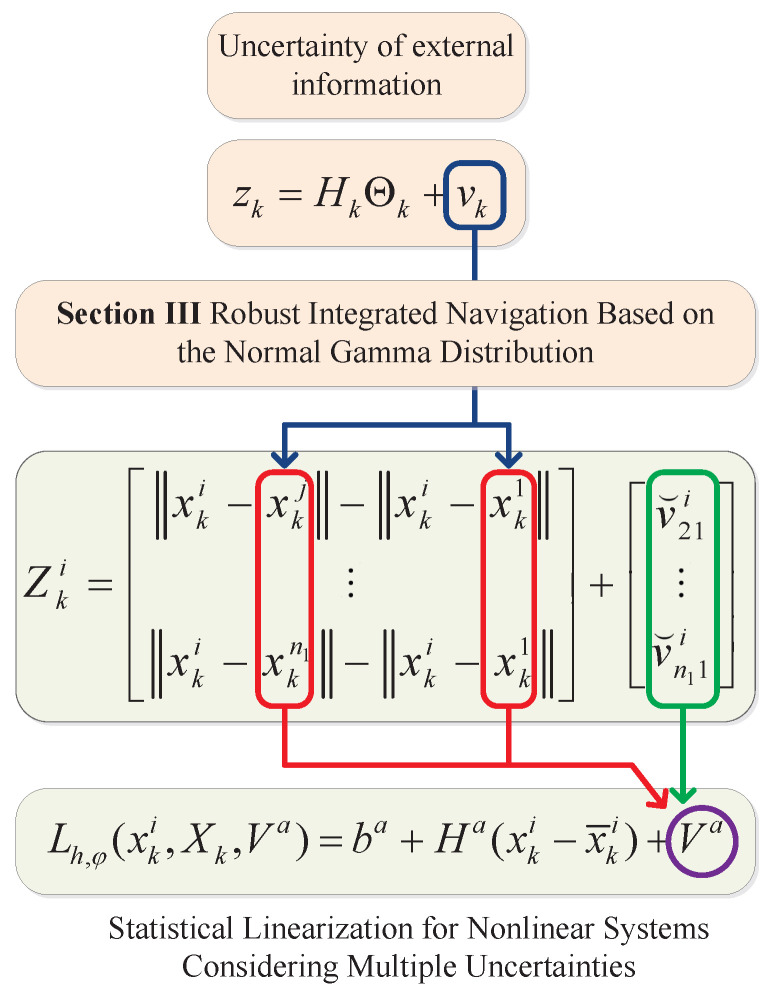
Illustration of the linearization method considering multiple uncertainties.

**Remark 1.** *(a) By performing deterministic sampling on $X_{k}$ and ${\overset{ˇ}{V}}_{k}^{i}$, both types of uncertainties are considered and equivalently represented as noise $V_{k}^{i}$, as illustrated by Figure 3.*

*(b) Since the elements of ${\overset{ˇ}{V}}_{k}^{i}$ follow a Gaussian distribution, unscented transformation for Gaussian distributions can be used for sampling. When sampling $X_{k}$, its posterior distribution is a conditional Gaussian distribution. To leverage the extensive results of fixed-point sampling for Gaussian distributions, the following approximation can be made for $x_{k}^{j}$:*

(58) $$p\left(x_{k}^{j}|\tau_{k}^{j}\right)=N\left(x_{k}^{j};{\hat{x}}_{k|k}^{j},\frac{P_{k|k}^{j}}{\tau_{k}^{j}}\right)\approx N\left(x_{k}^{j};{\hat{x}}_{k|k}^{j},\frac{P_{k|k}^{j}}{{\hat{\tau }}_{k|k}^{j}}\right)$$

## 5. Augmented Nonlinear Least Squares Estimation for Collaborative Positioning Among UAVs

For linear systems, the linear least squares method minimizes the fitting error,

(59) $$J={\left(z-Hx\right)}^{T}W\left(z-Hx\right)$$

and we set the gradient of *J* to zero to get

(60) $$\hat{x}={\left(H^{T}WH\right)}^{-1}H^{T}Wz$$

However, for nonlinear systems, using the above linear-structured estimator leads to an inherent mismatch. When the system is linear, the estimator is optimal. However, when the system is nonlinear, the linear structure of the estimator limits its potential for further performance improvement.

Recently proposed uncorrelated conversion methods [20,27] aim to find nonlinear functions of measurements and obtain filters with nonlinear structures. Their basic idea is to seek nonlinear and uncorrelated conversions g(z) of the original measurement *z* and then augment y=g(z) with *z* to get

(61) $$Z^{a}=\left[\begin{array}{c} Z\\ Y \end{array}\right]=\left[\begin{array}{c} h(x_{k}^{i},X_{k}^{j},{\overset{ˇ}{V}}^{i})\\ g(h\left(x_{k}^{i},X_{k}^{j},{\overset{ˇ}{V}}^{i}\right)) \end{array}\right]$$

The estimate is performed based on the augmented measurement $Z^{a}$. Using the statistical linearization method proposed in Section 4,

(62) $$Z^{a}\approx L_{h,\varphi }\left(x_{k}^{i},X_{k},V^{a}\right)=b^{a}+H^{a}\left(x_{k}^{i}-{\bar{x}}_{k}^{i}\right)+V^{a}$$

where

(63) $$b^{a}=\left[\begin{array}{c} h({\bar{x}}_{k}^{i},{\hat{X}}_{k|k},0)\\ g(h\left({\bar{x}}_{k}^{i},{\hat{X}}_{k|k},0\right)) \end{array}\right],H^{a}=\left[\begin{array}{c} H\\ H_{y} \end{array}\right],V^{a}=\left[\begin{array}{c} V^{i}\\ V^{Y} \end{array}\right]$$

with

(64) $$\begin{array}{ccc} & & H_{y}\\ & = & {\left.\frac{\partial g(h\left(x_{k}^{i},X_{k}^{j},{\overset{ˇ}{V}}^{i}\right))}{\partial h}\frac{\partial h(x_{k}^{i},X_{k}^{j},{\overset{ˇ}{V}}^{i})}{\partial x_{k}^{i}}\right|}_{x_{k}^{i}={\bar{x}}_{k}^{i},X_{k}={\hat{X}}_{k|k},{\overset{ˇ}{V}}_{k}^{i}=0} \end{array}$$

and

(65) $$\begin{array}{cc} E[V^{Y}]= & E[g\left(h\left({\bar{x}}_{k}^{i},X_{k},{\overset{ˇ}{V}}_{k}^{i}\right)\right)-g\left(h\left({\bar{x}}_{k}^{i},{\hat{X}}_{k|k},0\right)\right)] \end{array}$$

(66) $$\begin{array}{cc} \mathrm{Cov}[V^{Y}]= & E[g\left(h\left({\bar{x}}_{k}^{i},X_{k},{\overset{ˇ}{V}}_{k}^{i}\right)\right)-g\left(h\left({\bar{x}}_{k}^{i},{\hat{X}}_{k|k},0\right)\right){\left(\cdot \right)}^{T}] \end{array}$$

The calculation of these moments can be performed using the deterministic sampling method described in Equations (56) and (57), and also, the approximation in Equation (58) accounts for the uncertainty in sensor position estimation.

The location estimation of the remaining UAVs can be obtained by replacing the parameters in Equation (60) with those defined by (61)–(66). The following augmented nonlinear least squares (ANLS) estimation method is proposed. It is proven that the nonlinear estimate ${\hat{x}}^{\mathrm{ANLS}}$ based on the augmented measurement $z^{a}$ is more accurate than the original one ${\hat{x}}^{\mathrm{LLS}}$, that is, the mean square error of the augmented one is smaller than the original one,

(67) $$P_{{\hat{x}}^{\mathrm{ANLS}}}={\left[{\left(H^{a}\right)}^{T}W^{a}H^{a}\right]}^{-1}=P_{{\hat{x}}^{\mathrm{LLS}}}-P_{x}^{a}$$

where $P_{x}^{a}$ is a positive semi-definite matrix and, therefore, $P_{{\hat{x}}^{\mathrm{ANLS}}}$ is smaller than $P_{{\hat{x}}^{\mathrm{LLS}}}$.

Following [20], the uncorrelated conversion function is selected as

(68) $$Y=g\left(Z\right)=\left[\begin{array}{c} {\left(z_{21}+r_{1}\right)}^{2}\\ \vdots \\ {\left(z_{n_{1}}+r_{1}\right)}^{2} \end{array}\right]$$

Using the the augmented measurement (61) and based on the TWLS method, the collaborative positioning among UAVs is achieved. Detailed steps are summarized as follows.

### 5.1. First Step

Based on the statistical linearization model (62), the parameters defined by (63)–(66) can be calculated by

(69) $$Y=\left[\begin{array}{c} \frac{1}{2}r_{21}^{2}\\ \vdots \\ \frac{1}{2}r_{n_{1}1}^{2} \end{array}\right],$$

(70) $$b_{Y}=\left[\begin{array}{c} \frac{1}{2}\left({\left({\hat{x}}_{k|k}^{2}\right)}^{T}{\hat{x}}_{k|k}^{2}-{\left({\hat{x}}_{k|k}^{1}\right)}^{T}{\hat{x}}_{k|k}^{1}\right)\\ \vdots \\ \frac{1}{2}\left({\left({\hat{x}}_{k|k}^{n_{1}}\right)}^{T}{\hat{x}}_{k|k}^{n_{1}}-{\left({\hat{x}}_{k|k}^{1}\right)}^{T}{\hat{x}}_{k|k}^{1}\right) \end{array}\right]$$

(71) $$\begin{array}{cc} H_{Y}= & \left[\begin{array}{cc} {\left({\hat{x}}_{k|k}^{2}-{\hat{x}}_{k|k}^{1}\right)}^{T} & r_{21}\\ \vdots & \vdots \\ {\left({\hat{x}}_{k|k}^{n_{1}}-{\hat{x}}_{k|k}^{1}\right)}^{T} & r_{n_{1}1} \end{array}\right], \end{array}$$

(72) $$\begin{array}{cc} V^{Y}= & \left[\begin{array}{c} r_{21}v_{21}-\frac{1}{2}v_{21}^{2}\\ \vdots \\ r_{n_{1}1}v_{n_{1}1}-\frac{1}{2}v_{n_{1}1}^{2} \end{array}\right] \end{array}$$

The moments ${\bar{V}}^{a}$ and $C_{V^{a}}$ of $V^{Y}$ can be obtained by deterministic sampling. The estimate is given by

(73) $$\begin{array}{cc} \hat{u}= & {\left[{\left(H^{a}\right)}^{T}W^{a}H^{a}\right]}^{-1}{\left(H^{a}\right)}^{T}W^{a}\left(Z^{a}-b^{a}-{\bar{V}}^{a}\right) \end{array}$$

(74) $$\begin{array}{cc} P_{\hat{u}}= & {\left[{\left(H^{a}\right)}^{T}W^{a}H^{a}\right]}^{-1} \end{array}$$

with $\hat{u}=\left[\begin{array}{c} {\overset{ˇ}{x}}_{k|k}^{i}\\ {\hat{r}}_{1} \end{array}\right]$, which also estimates the range $r_{1}$.

### 5.2. Second Step

According to TWLS, based on $\hat{u}$ and the relationship between $r_{1}$ and $x^{i}$, the estimate of $x^{i}$ can be further improved. The statistical linearization model is constructed as follows,

(75) $$z^{\prime }=H^{\prime }u^{\prime }+v^{\prime }$$

where

(76) $$\begin{array}{cc} u^{\prime }= & \left[\left(x_{k}^{i}-{\hat{x}}_{k|k}^{j}\right)⊙\left(x_{k}^{i}-{\hat{x}}_{k|k}^{j}\right)\right],H^{\prime }=\left[\begin{array}{c} I_{3\times 3}\\ 1_{1\times 3} \end{array}\right] \end{array}$$

(77) $$\begin{array}{cc} z^{\prime }= & \left[\begin{array}{c} \left({\overset{ˇ}{x}}_{k|k}^{i}-{\hat{x}}_{k|k}^{j}\right)⊙\left({\overset{ˇ}{x}}_{k|k}^{i}-{\hat{x}}_{k|k}^{j}\right)\\ {\hat{r}}_{1} \end{array}\right] \end{array}$$

(78) $$\begin{array}{cc} v^{\prime }= & \left[\begin{array}{c} 2\left({\overset{ˇ}{x}}_{k|k}^{i}-x_{k}^{j}\right)⊙{\tilde{x}}_{k|k}^{i}+{\tilde{x}}_{k|k}^{i}⊙{\tilde{x}}_{k|k}^{i}\\ 2r_{1}{\tilde{r}}_{1}+{\tilde{r}}_{1}{\tilde{r}}_{1} \end{array}\right] \end{array}$$

with ${\tilde{x}}_{k|k}^{i}=x_{k}^{i}-{\overset{ˇ}{x}}_{k|k}^{i}$, ${\tilde{r}}_{1}=r_{1}-{\hat{r}}_{1}$, and ${\left[a_{1}\;a_{2}\;a_{3}\right]}^{T}⊙{\left[b_{1}\;b_{2}\;b_{3}\right]}^{T}={\left[a_{1}b_{1}\;a_{2}b_{2}\;a_{3}b_{3}\right]}^{T}$. The estimate of $\tilde{u}$ is given by

(79) $${\hat{u}}_{k|k}^{\prime }={\left[{\left(\tilde{H}\right)}^{T}\tilde{W}\tilde{H}\right]}^{-1}{\left(\tilde{H}\right)}^{T}\tilde{W}\left(\tilde{z}-E\left[\tilde{v}\right]\right)$$

with E$\left[\tilde{v}\right]$ obtained by deterministic sampling.

Finally, the location of the *i*th UAV is

(80) $${\hat{x}}_{k|k}^{i}=U{\left({\hat{u}}_{k|k}^{\prime }\right)}^{1/2}+{\hat{x}}_{k|k}^{j}$$

where U= diag(sign(${\hat{u}}_{k|k}^{\prime }-{\hat{x}}_{k|k}^{j}$)) is a sign function.

The steps for cooperative localization in a swarm of UAVs can be summarized as follows. Firstly, the robust filter for integrated navigation based on the normal gamma distribution (RINNG) method described in Section 3 is utilized to estimate the error states, primarily addressing the uncertainty in measurement quality caused by outliers in external correction information. Based on this external correction information and the INS resolution, integrated navigation is performed to determine the positions of a subset of UAVs. Using these position estimates and estimates of their uncertainties, the statistical linearization of the distance measurement model is then carried out using the method outlined in Section 4. Finally, based on the linearized model, the augmented nonlinear least squares (ANLS) method is applied to estimate the positions of the other UAVs, ultimately yielding the localization results for the entire UAV swarm.

## 6. Simulation

The simulation considers three scenarios: The first scenario involves positioning using external correction information corrupted by outliers, primarily for evaluating the proposed robust filter for integrated navigation based on the normal gamma distribution (RINNG) method. The second scenario involves TDOA positioning among UAVs, primarily for evaluating the proposed augmented nonlinear least squares (ANLS) method. The third scenario is collaborative positioning of a swarm of UAVs, utilizing a unified scenario to comprehensively evaluate the methods proposed in this paper.

### 6.1. Scenario 1: Positioning Using Outlier Corrupted External Correction Measurements

Assume that the target has a constant-turn motion,

(81) $$x_{k+1}=\left[\begin{array}{ccccc} 1 & \frac{\sin\omega T}{\omega } & 0 & \frac{\cos\omega T}{\omega } & 0\\ 0 & \cos\omega T & 0 & -\sin\omega T & 0\\ 0 & \frac{1-\cos\omega T}{\omega } & 1 & \frac{\sin\omega T}{\omega } & 0\\ 0 & \sin\omega T & 0 & \cos\omega T & 0\\ 0 & 0 & 0 & 0 & 1 \end{array}\right]x_{k}+w_{k}$$

where $x_{k}={\left[x_{k},{\dot{x}}_{k},y_{k},{\dot{y}}_{k},\omega \right]}^{T}$ is the state vector that consists of position, velocity, and the turn rate, the sampling interval T=1s, the process noise $w_{k}∼N\left(0,Q_{k}\right)$ with $Q_{k}=$ diag$\left(q_{1}^{2}M,q_{1}^{2}M,q_{2}^{2}T\right)$, $q_{1}=0.1$ m/s^2^ , $q_{2}=1.74\times 10^{-4}$, and

(82) $$M=\left[\begin{array}{cc} T^{3}/3 & T^{2}/2\\ T^{2}/2 & T \end{array}\right]$$

The model of range and bearing measurements is

(83) $$z_{k}=\left[\begin{array}{c} \sqrt{x_{k}^{2}+y_{k}^{2}}\\ \arctan\frac{y_{k}}{x_{x}} \end{array}\right]+v_{k}$$

where $p\left(v_{k}\right)=0.9N\left(0,R\right)+0.1N\left(0,100R\right)$ with covariance R=diag$\left(\sigma_{r}^{2},\sigma_{\theta }^{2}\right)$ ($\sigma_{r}=10$ m, $\sigma_{\theta }=\sqrt{10}$ mrad). The probability of outlier occurrence is 0.1. When an outlier appears, the measurement noise covariance becomes 100 times that under normal conditions.

**Figure 4:**
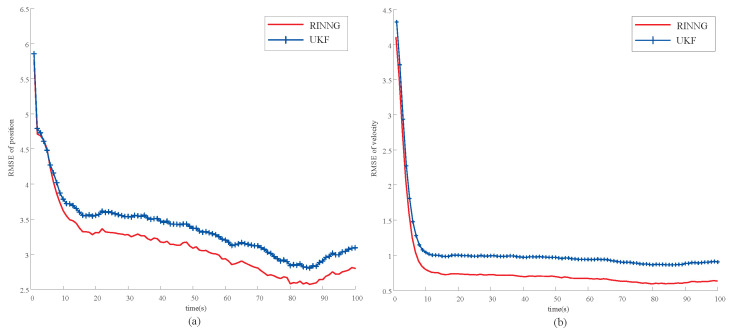
Simulation scenario 1: positioning using outlier corrupted external correction measurements. (**a**) RMSE of position estimation; (**b**) RMSE of velocity estimation.

Figure 4 shows the RMSEs of position and of velocity over 5000 Monte Carlo runs. As can be seen from the figure, the proposed RINNG method outperforms the UKF [26] in both position and velocity estimation, effectively addressing the challenge of outliers present in external correction information.

### 6.2. Scenario 2: Mutual Positioning Using TDOA Measurements

**Figure 5:**
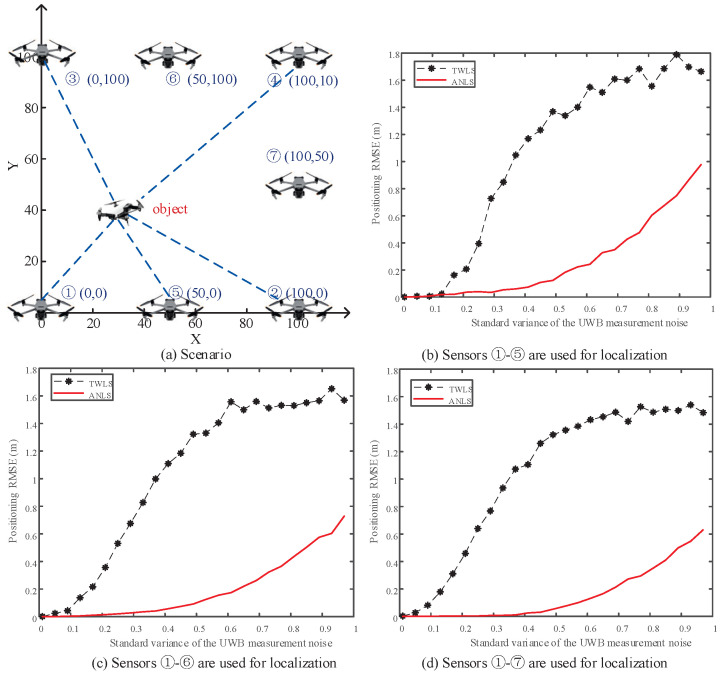
Simulation scenario 2: mutual positioning using TDOA measurements. (**a**) The scenario; (**b**) positioning RMSE based on 5 sensors; (**c**) positioning RMSE based on 6 sensors; (**d**) positioning RMSE based on 7 sensors.

Consider the TDOA localization scenario depicted in Figure 5a, where the positions of UAVs numbered 1 to 7 are known (as annotated in the figure), serving as sensors to locate an object with an unknown position. Compare the localization accuracy of the proposed ANLS and the classical TWLS [16] methods under different numbers of sensors and noise levels. Figure 5b, c, and d, respectively, present the localization errors from 1000 Monte Carlo simulations using 5,6, and 7 UAVs for positioning. In each figure, the horizontal axis represents the standard variance of measurement noise, and the vertical axis represents the positioning RMSE. It can be observed from the figures that with more sensors, the localization errors of both TWLS and ANLS decrease. When using the same number of sensors, larger noise results in poorer localization accuracy. In all scenarios, the proposed ANLS method significantly improves the localization accuracy compared to TWLS, with a reduction in localization error by more than 40%, validating the effectiveness of the proposed ANLS method.

### 6.3. Scenario 3: UAV Swarm Collaborative Positioning

**Figure 6:**
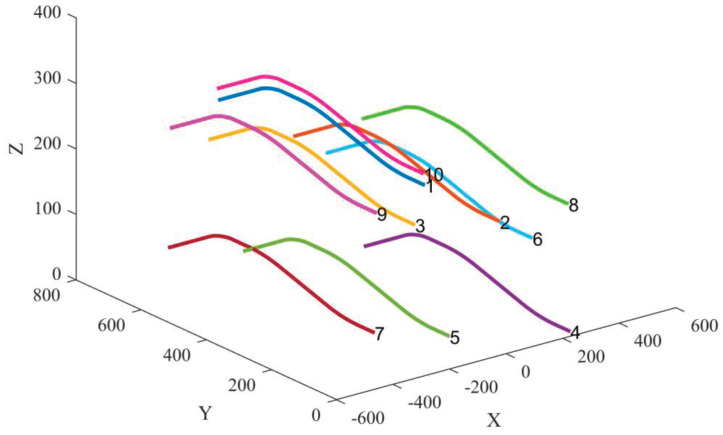
Simulation scenario 3: 10 UAVs are considered for collaborative integrated navigation, where 5 of them are equipped with high precision INSs and capable of receiving external correction information.

Consider the UAV swarm collaborative positioning scenario shown in Figure 6. The figure illustrates the true trajectories of the UAVs, with the labeled points indicating the starting positions of their flights. In the UAV swarm, UAVs labeled 1–5 are equipped with high-precision INSs and are assumed to receive external correction information. UAVs labeled 6–10 are equipped with low-precision INSs and rely on TDOA positioning with the assistance of UAVs 1–5 to improve their navigation accuracy. The error parameters of the INS for each UAV are summarized in Table 1. As shown in the table, the INS parameters of UAVs 1–5 are identical, while the INS performance of UAVs 6–10 is inferior to that of UAVs 1–5, with varying values among them.

**Figure 7:**
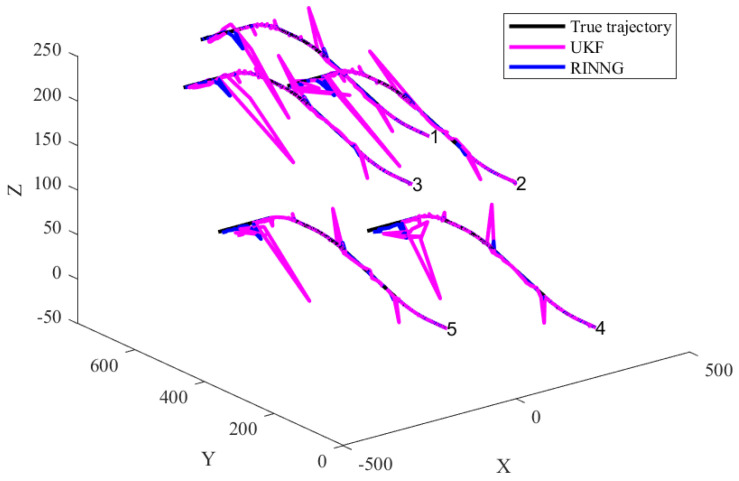
Comparison of integrated navigation results.

Figure 7 illustrates the self-localization results of integrated navigation for UAVs 1–5 when external correction information is corrupted by outliers. It is assumed that, in the absence of interference, the external correction information is highly accurate, with a measurement noise covariance of $R_{k}=$ diag$\left(10^{-10},10^{-10},10^{-4}\right)$. However, each UAV has a 0.05 probability of receiving outliers in the correction information, in which case the measurement noise covariance increases to $10^{6}R_{k}$. As shown in the figure, the presence of outliers significantly impacts positioning accuracy. The UKF method produces fused localization results with substantial errors. In contrast, the proposed RINNG method, which accounts for the presence of outliers and adaptively adjusts the covariance of measurement noise, effectively mitigates the impact of outliers and provides robust self-localization results.

Table 2 compares the RMSE of the positioning for UAVs 1–5 as provided by the UKF and RINNG methods. The results show that RINNG achieves significantly higher positioning accuracy than UKF, demonstrating its robustness in handling outliers in external correction data.

**Figure 8:**
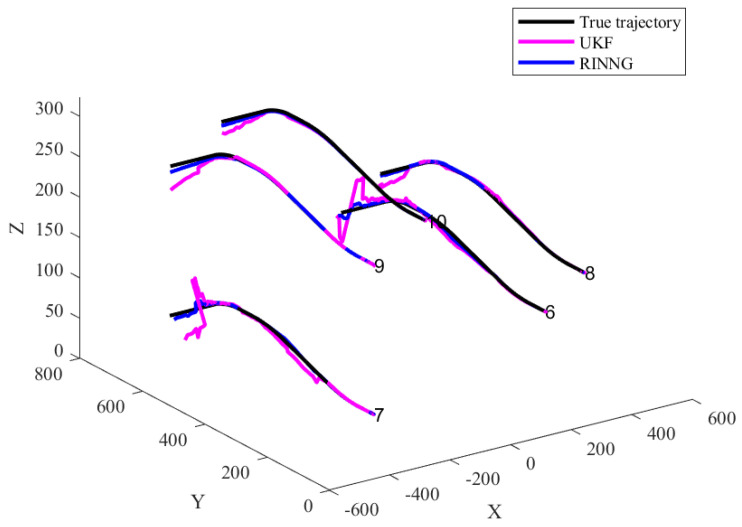
Comparison of collaborative positioning results.

Figure 8 shows the results of mutual positioning for UAVs 6–10, based on the self-localization results of UAVs 1–5, which serve as sensors. Due to the influence of outliers in external correction data, the UKF self-localization results exhibit significant errors. Building mutual positioning on this basis further propagates these errors, causing the positioning deviations of UAVs 6–10 to increase over time, eventually leading to divergence. The proposed statistical linearization model accounts for the uncertainties in self-localization, ensuring that the positioning results of UAVs 6–10 do not diverge over time.

**Figure 9:**
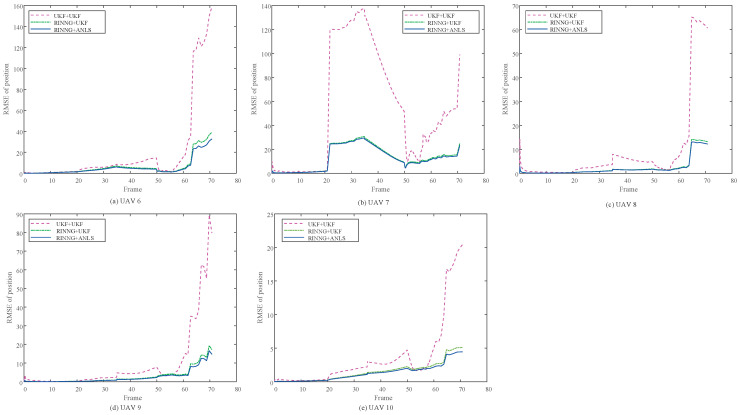
RMSEs of collaborative positioning.

Figure 9 shows the RMSE of the positioning for each of the UAVs 6–10. As observed, due to insufficient consideration of various uncertainties, the positioning results provided by the UKF method [26] diverge in the later stages. In contrast, the proposed method adequately accounts for these uncertainties, suppressing their impact on positioning accuracy and keeping navigation errors within an acceptable range. The figure also presents results obtained using only RINNG without ANLS. It is evident that incorporating ANLS further improves the least squares positioning accuracy.

Table 3 summarizes the performance of UAVs 6–10 and presents an ablation study of the proposed method. The localization of UAVs 6–10 is conducted in two steps: first, locating UAVs 1–5, and second, locating UAVs 6–10 based on the localization results of UAVs 1–5. In the table, UKF+UKF indicates that both steps are based on the UKF. RINNG+UKF indicates that the proposed RINNG is used for locating UAVs 1–5 to handle outliers, while UKF is employed in the second step. RINNG+ANLS indicates that both steps utilize the proposed methods. As shown in the table, the RINNG method demonstrates remarkable performance in handling outliers, while the ANLS method further improves localization accuracy in mutual localization.

The above results validate the effectiveness of the proposed method. The proposed RINNG method can effectively handle outliers in external correction data, while the statistical linearization approach accounts for the uncertainties in self-localization results. Additionally, the ANLS method further enhances positioning accuracy for nonlinear localization problems.

## 7. Conclusions

This paper investigates the multiple uncertainties present in cooperative navigation and positioning for UAV swarms. It primarily addresses challenges such as the uncertainty arising from outliers in external correction information, the uncertainty in the self-position estimation of leaders engaged in mutual localization within the swarm, and incomplete information processing due to the strong nonlinearity of mutual localization measurements. The basic idea of the proposed method is to quantify the primary source of uncertainty, namely the uncertainty in external correction information, using gamma variables. A statistical linearization method for nonlinear models incorporating gamma variables is then proposed. Based on the linearized model, the paper further applies an uncorrelated conversion method to realize nonlinear least squares positioning. When dealing with uncertainties as a whole, the modeling and evolution of uncertainties are considered in an integrated manner. Based on the proposed method, robust navigation and positioning for the entire UAV swarm can be achieved.

Simulation verifications were conducted in three scenarios. The proposed robust integrated navigation method effectively suppressed large positioning deviations caused by outliers. For mutual localization within the swarm, the proposed statistical linearization method and the least squares method based on augmented data effectively considered the uncertainty in leader positioning, enabling further utilization of nonlinear measurement information and achieving higher positioning accuracy. The proposed methods can be applied not only to cooperative navigation of UAV swarms but also to navigation and positioning in other multi-UAV system collaborations.

## Figures and Tables

**Table 1:** Parameter settings of INSs of UAVs.

No.	Accelerometer (μg)		Gyroscope (^∘^/h)	
	Const. Drift	Rand. Drift	Const. Drift	Rand. Drift
1–5	10	100	1	1
6	100	10,000	10	10
7	10	10,000	10	10
8	10	1000	1	10
9	10	1000	10	15
10	100	1000	10	15

**Table 2:** Time-averaged RMSE of UAVs 1–5.

No.	1	2	3	4	5
UKF	38.0593	47.2109	47.0874	44.5690	58.7933
RINNG	3.5439	3.9141	3.9060	3.8053	4.3767

**Table 3:** Time-averaged RMSE of UAVs 6–10 and an ablation study.

No.	6	7	8	9	10
UKF+UKF	6.2566	24.0521	7.6684	6.3815	4.7194
RINNG+UKF	2.5273	10.3385	2.7722	3.8936	2.7534
RINNG+ANLS	2.2128	9.5732	2.6567	3.3472	2.3087

## Data Availability

Data are contained within the article.

## Abbreviations

The following abbreviations are used in this manuscript:

UAV	unmanned aerial vehicle
GNSS	global navigation satellite system
NLOS	non-line-of-sight
INS	inertial navigation system
SLAM	simultaneous localization and mapping
UWB	ultra wide band
PDF	probability density function
TDOA	time difference of arrival
TWLS	two-step weighted least squares
RINNG	robut integrated navigation based on normal gamma distribution
RMSE	root mean square error
UKF	unscented Kalman filter
ANLS	augmented nonlinear least squares

## References

[1] Tahir A., Boing J., Haghbayan M.H., Toivonen H.T., Plosila J. (2019). Swarms of Unmanned Aerial Vehicles—A Survey. J. Ind. Inf. Integr..

[2] Sun L., Zhang J., Yang Z., Fan B. (2023). A motion-aware siamese framework for unmanned aerial vehicle tracking. Drones.

[3] Li Z., Jiang C., Gu X., Xu Y., Cui J. (2024). Collaborative positioning for swarms: A brief survey of vision, LiDAR and wireless sensors based methods. Def. Technol..

[4] Elamin A., Abdelaziz N., El-Rabbany A. (2022). A GNSS/INS/LiDAR integration scheme for UAV-based navigation in GNSS-challenging environments. Sensors.

[5] Mostafa M., Zahran S., Moussa A., El-Sheimy N., Sesay A. (2018). Radar and visual odometry integrated system aided navigation for UAVS in GNSS denied environment. Sensors.

[6] Lee J.C., Chen C.C., Shen C.T., Lai Y.C. (2022). Landmark-based scale estimation and correction of visual inertial odometry for vtol uavs in a gps-denied environment. Sensors.

[7] Negru S.A., Geragersian P., Petrunin I., Guo W. (2024). Resilient Multi-Sensor UAV Navigation with a Hybrid Federated Fusion Architecture. Sensors.

[8] Tavares A.J., Oliveira N.M. (2024). A Novel Approach for Kalman Filter Tuning for Direct and Indirect Inertial Navigation System/Global Navigation Satellite System Integration. Sensors.

[9] Konovalenko I., Kuznetsova E., Miller A., Miller B., Popov A., Shepelev D., Stepanyan K. (2018). New approaches to the integration of navigation systems for autonomous unmanned vehicles (UAV). Sensors.

[10] Zhang L., Lan J., Li X.R. A normal-Gamma filter for linear systems with heavy-tailed measurement noise. Proceedings of the 21th International Conference on Information Fusion.

[11] Qi Y., Zhong Y., Shi Z. (2020). Cooperative 3-D relative localization for UAV swarm by fusing UWB with IMU and GPS. J. Phys. Conf. Ser..

[12] Belfadel D., Haessig D., Chibane C. (2024). Range-Only EKF-Based Relative Navigation for UAV Swarms in GPS-Denied Zones. IEEE Access.

[13] Chen H., Xian-Bo W., Liu J., Wang J., Ye W. (2020). Collaborative multiple UAVs navigation with GPS/INS/UWB jammers using sigma point belief propagation. IEEE Access.

[14] Chen M., Xiong Z., Song F., Xiong J., Wang R. (2022). Cooperative navigation for low-cost UAV swarm based on sigma point belief propagation. Remote Sens..

[15] Zhu X., Lai J., Chen S. (2022). Cooperative Location Method for Leader-Follower UAV Formation Based on Follower UAV’s Moving Vector. Sensors.

[16] Chan Y.T., Ho K.C. (1994). A simple and efficient estimator for hyperbolic location. IEEE Trans. Signal Process..

[17] Sun L., Ji B., Lan J., He Z., Pu J. (2017). Tracking of maneuvering complex extended object with coupled motion kinematics and extension dynamics using range extent measurements. Sensors.

[18] Xu Z., Zhou G. (2023). Long-Time Coherent integration for radar detection of maneuvering targets based on accurate range evolution model. IET Radar Sonar Navig..

[19] Xu L., Liang Y., Duan Z., Zhou G. (2019). Route-based dynamics modeling and tracking with application to air traffic surveillance. IEEE Trans. Intell. Transp. Syst..

[20] Li Q., Lan J., Zhang L., Chen B., Zhu K. (2021). Augmented nonlinear least squares estimation with applications to localization. IEEE Trans. Aerosp. Electron. Syst..

[21] Zhang L., Lan J., Li X.R. Normal-Gamma IMM filter for linear systems with non-Gaussian measurement noise. Proceedings of the 22th International Conference on Information Fusion.

[22] Agamennoni G., Nieto J.I., Nebot E.M. (2012). Approximate inference in state-space models with heavy-tailed noise. IEEE Trans. Signal Process..

[23] Särkkä S., Nummenmaa A. (2009). Recursive noise adaptive Kalman filtering by variational Bayesian approximations. IEEE Trans. Autom. Control.

[24] Zhang L., Lan J. (2021). Tracking of extended object using random matrix with non-uniformly distributed measurements. IEEE Trans. Signal Process..

[25] Arasaratnam I., Haykin S., Elliott R.J. (2007). Discrete-time nonlinear filtering algorithms using Gauss–Hermite quadrature. Proc. IEEE.

[26] Julier S.J., Uhlmann J.K. (2004). Unscented filtering and nonlinear estimation. Proc. IEEE.

[27] Lan J., Li X.R. (2015). Nonlinear estimation by LMMSE-based estimation with optimized uncorrelated augmentation. IEEE Trans. Signal Process..

